# Incomplete Bilateral Caroticoclinoid Foramina in a Dry Human Skull: A Rare Case Reported With Clinicoanatomical Relevance

**DOI:** 10.7759/cureus.115256

**Published:** 2026-08-26

**Authors:** Keerthy Ajay Kumar, Tarun Maheshwari, Rajat S Das

**Affiliations:** 1 Anatomy, All India Institute of Medical Sciences, Raebareli, Raebareli, IND

**Keywords:** anterior clinoid process, caroticoclinoid foramen, caroticoclinoid ligament, clinoidectomy, internal carotid artery (ica)

## Abstract

Caroticoclinoid foramen (CCF) is an inconsistent structure formed due to the ossification of either the caroticoclinoid ligament or a dural fold extending between the anterior and middle clinoid processes of the sphenoid bone during development. For the surgical procedure of anterior clinoidectomy in case of a paraclinoid aneurysm of the internal carotid artery (ICA), the presence of a bony CCF may lead to heavy haemorrhage and other complications like CSF rhinorrhea, injury of adjacent structures like the optic nerve and oculomotor nerve. We present a case of incomplete bilateral caroticoclinoid foramina in a dry female human skull of unknown age, with no structural deformity. The diameter of the incomplete CCF on the right side measured 3.9 mm, while that of the left side was 4 mm. The interspicular distance between the opposing bony spurs was estimated to be 1.1 mm on the right side and 0.8 mm on the left side. The ICA, while traversing through the cavernous sinus, passes medial to the anterior clinoid process. Therefore, neurosurgeries approaching the anterior clinoid process are likely to damage the ICA and surrounding intracranial structures if CCF is present. This case is interesting due to the unusual pattern with regard to its bilaterality, not reported earlier, and its significance to the knowledge of the skull-base anatomy and implications for neurosurgical anatomy.

## Introduction

Embryologically, the caroticoclinoid foramen (CCF) is a congenital anatomical variant produced by ossification of the caroticoclinoid ligament (CCL) or the dural fold connecting the anterior and middle clinoid processes. Its occurrence in fetal and infant skulls indicates that it develops during chondrocranial ossification rather than representing an age-related degenerative change [1]. Based on morphology, Keyes described three forms of this foramen: a complete type, where a continuous bony bridge is present; a contact type, in which the anterior and middle clinoid processes meet through a sutural connection; and an incomplete type, characterized by partial bony projections that fail to unite [2]. As per the observations of Keyes, this foramen was identified in approximately 5-10% of examined dry skulls, with the incomplete variant being the most frequently encountered [2].

The internal carotid artery (ICA) ascends through the carotid canal to enter the cavernous sinus, where it lies in relation to the carotid sulcus. The anterior clinoid process (ACP) forms a structural roof over both the cavernous sinus and the paraclinoid segment of the ICA. When a CCF is present, it may surround the paraclinoid portion of the artery. After piercing the dura mater, the ICA turns back below the optic nerve [3]. Among the different forms, the incomplete type, especially when associated with sharp bony projections, poses the greatest risk, as it may injure the arterial wall [4].

Such anatomical variation has important clinical implications. In particular, an incomplete CCF can predispose to rupture of the ICA, potentially leading to the formation of a carotico-cavernous fistula, an abnormal communication between the artery and the cavernous sinus [4]. Furthermore, the presence of a fully ossified CCL may complicate surgical procedures involving anterior clinoidectomy, such as those performed for paraclinoid aneurysms, due to the risk of vascular injury and excessive hemorrhage [5]. It may also restrict mobilization of the ICA during surgical clipping of aneurysms arising from the clinoid segment or its branches, including the ophthalmic artery [6,7].

Additional caution is required during surgical drilling in the sellar and parasellar regions. Pneumatization of the ACP may increase the likelihood of injury to adjacent structures such as the optic nerve and ICA, and may also result in complications including cerebrospinal fluid (CSF) rhinorrhea and damage to the oculomotor nerve [8]. The ossified caroticoclinoid ligament can compress the cavernous sinus and ICA, which can cause headache and weakness of extraocular muscles due to the involvement of surrounding neurovascular structures [9,10].

Here, we report incomplete bilateral caroticoclinoid foramina in a dry female human skull of unknown age, which contributes to the current anatomical literature, especially from the point of view of the neurosurgical procedures in the parasellar region. This knowledge of uncommon incidence would certainly alert and help neurosurgeons for better preoperative planning.

## Case presentation

During routine osteological examination of an adult dry female human skull of unknown age obtained from an institutional collection, bilateral incomplete CCF were observed in the sphenoid bone. Sex was defined using cranial features like less prominent supraorbital ridges, sharper margins, and round shape of the orbits, vertical slope of the forehead with prominence in the frontal eminences. The skull was shorter in proportion to its breadth with prominent parietal eminences. The base of the skull was intact with no structural deformity. The CCF were formed between the ACP and middle clinoid process (MCP) of the sphenoid bone. The ACP was identified at the medial extremity of the lesser wing of the sphenoid adjacent to the optic canal, while the MCP projected from the lateral margin of the tuberculum sellae on both sides.

**Figure 1:**
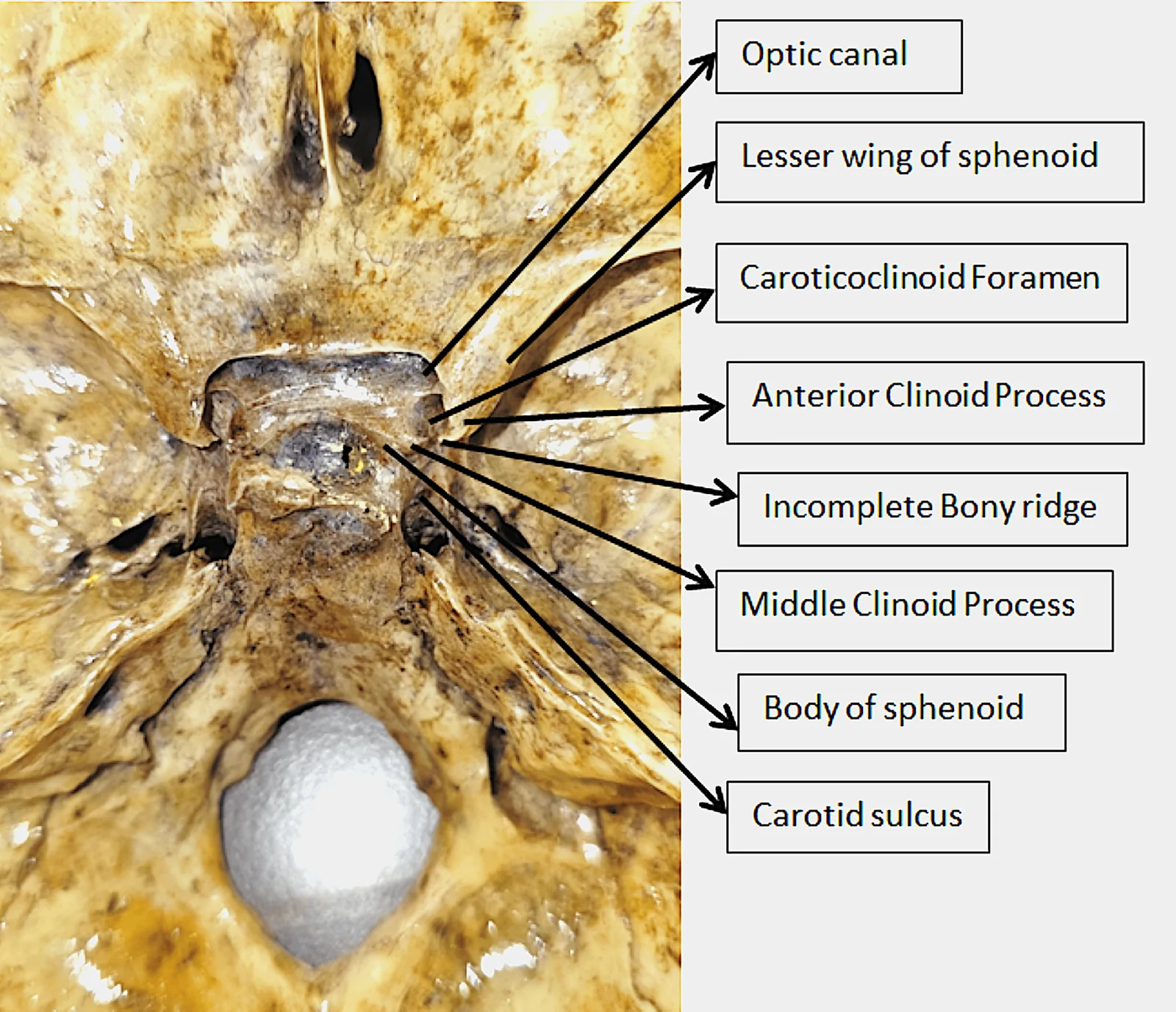
Interior of the skull showing bilateral incomplete caroticoclinoid foramina formed between the anterior clinoid process of the lesser wing of sphenoid and the middle clinoid process of the body of sphenoid.

On detailed inspection, osseous projections extended from both the ACP and MCP bilaterally towards each other. However, these bony extensions failed to unite completely, resulting in the formation of incomplete CCF on both sides. The intervening gap between the opposing bony spicules prevented the establishment of a complete osseous ring (Figure 1).

**Figure 2:**
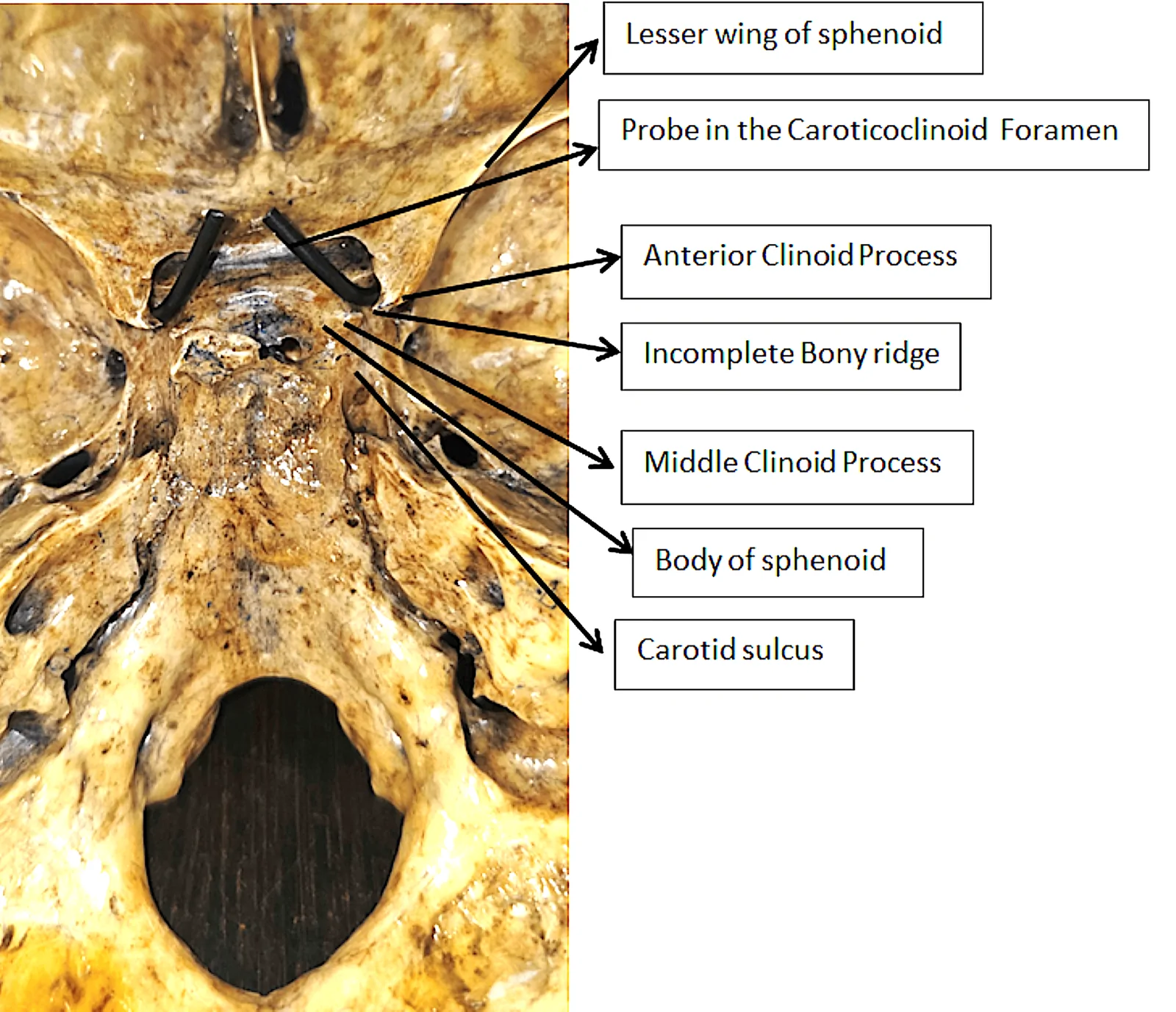
Interior of the skull showing probe in the incomplete caroticoclinoid foramina on both sides formed between the anterior clinoid process of the lesser wing of sphenoid and the middle clinoid process of the body of sphenoid.

The diameter of the incomplete CCF on the right side measured 3.9 mm, while that of the left side was 4 mm. The interspicular distance between the opposing bony spurs was 1.1 mm on the right side and 0.8 mm on the left side. The minimum distance between the tip of the incomplete caroticoclinoid bony ridge and the posterior margin of the optic canal was 6.8 mm on the right side and 6.2 mm on the left side, indicating the close anatomical relationship of the incomplete osseous bridge to the optic canal. Measurements were taken using a carbon fiber digital caliper with an accuracy of ±0.2mm and were repeated by two observers. Probes were placed in the incomplete CCF on each side (Figure 2).

**Figure 3:**
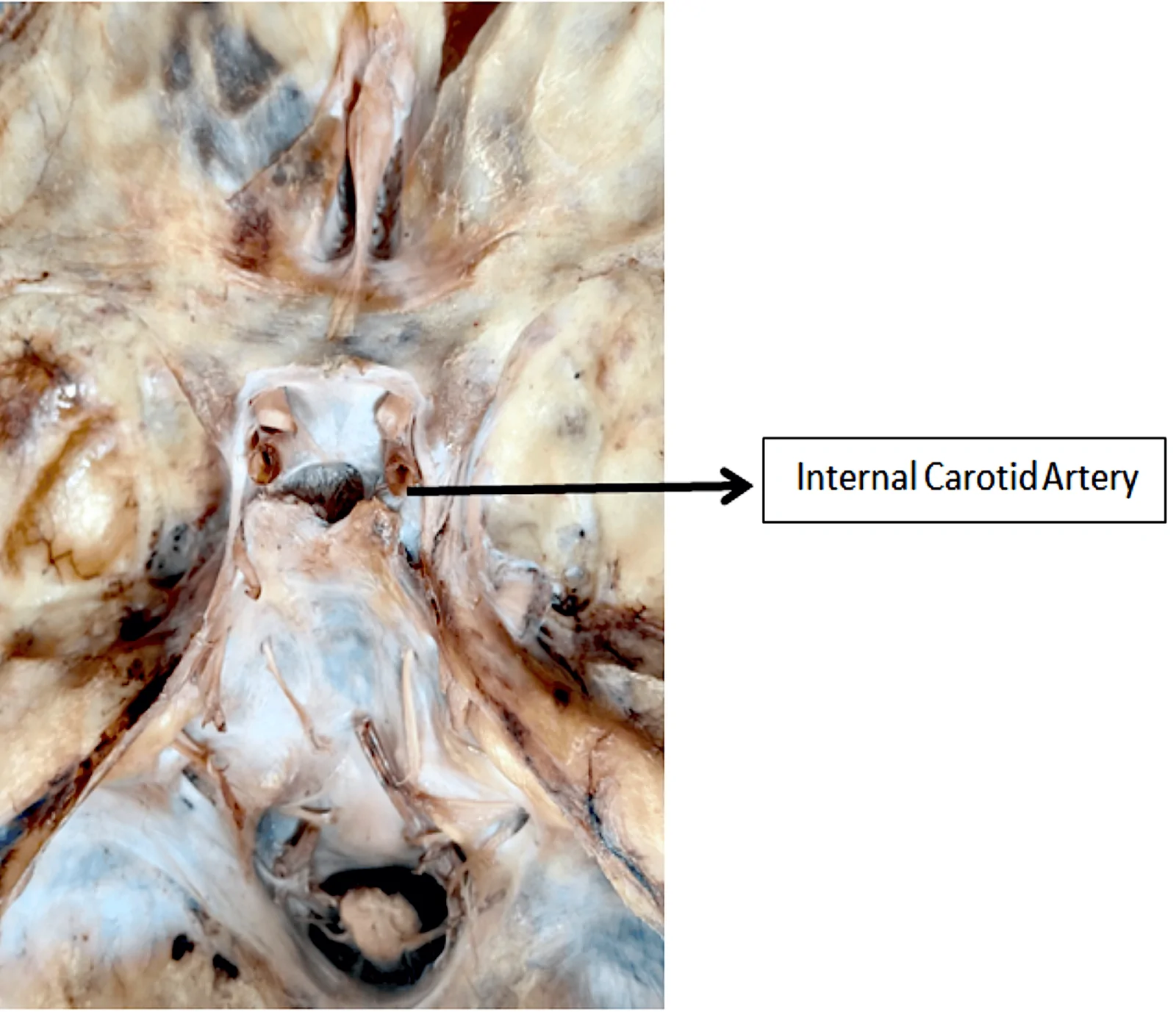
Interior of cranium of an wet specimen (reference for comparison) showing the regular position of the internal carotid artery medial to the anterior clinoid process.

As a reference, in a separate anatomical wet specimen without cranial abnormalities or osseous variations dissected during the routine practical classes, the ICA was observed coursing medial to the anterior clinoid process in the cavernous sinus region, which is its usual relation to the medial clinoid process (Figure 3).

In the event of incomplete incidence, such as in the present case, the free edges of the CCF lie in close proximity to the region occupied by the paraclinoid segment of the ICA, thereby increasing the risk of neurovascular complications and posing challenges during skull-base and parasellar surgical procedures.

## Discussion

Keyes examined a total of 2187 skulls, of which a complete foramen was present in 5.35%, the contact type in 0.32%, and the incomplete type in 10% [2]. Research indicates that environmental factors, terrain, and race all affect the incidence of CCF. Japanese, Portuguese, Germans, Koreans, and Brazilians presented with low incidence in comparison to Indians, whereas Sardanians, Caucasians, and Turks presented with high incidence [8-14].

According to a previous study, CCF nearly invariably changes the morphology of ICA, and these changes have the potential to compress the cavernous sinus and cause serious neurological issues [15]. On CT angiography, the ossified caroticoclinoid ligament may appear as a para-posterior communicating artery aneurysm due to traction on the ICA, which can cause a severe headache [16]. The ICA may burst as a result of severe manipulation and drilling of the ossified ligament during anterior clinoidectomy, which is necessary for procedures like clinoidal segment aneurysm or the removal of a central skull base tumor, which exposes the ICA and cavernous sinuses [17]. The ossified CCL may obstruct the MCP presentation, which serves as a landmark for the anteromedial side of the cavernous sinus and for the passage of ICA from its clinoidal segment to its cavernous part, making the endonasal approach to the pituitary gland more difficult during endoscopic treatments [18]. Surgeons should have precise knowledge about this variant while performing optic nerve decompression, which involves removal of the ACP [19]. 

In a study of 100 dry skulls by Muthukumar et al., 3% of female skulls and 5% of male skulls showed the presence of bilateral incomplete CCF [20]. Desai et al. studied 223 dry human skulls and reported that 3% of skulls had bilateral incomplete CCF [21]. Even though case reports of complete CCF have been reported, no isolated case reports of bilateral incomplete CCF have been documented in previous literature to the best of our knowledge. In the present case, the observed bilateral incomplete CCF likely resulted from partial ossification of the caroticoclinoid ligament or dural fold. The associated bony spicules extending from the anterior and middle clinoid processes may pose a risk to the ICA, particularly due to its pulsatile nature or under conditions of elevated arterial pressure. Therefore, preoperative evaluation of CCF using digital radiography or CT imaging is essential for safer surgical planning in the sellar region.

## Conclusions

The ICA, while traversing through the cavernous sinus, passes medial to the ACP. The bony spicule in the incomplete type of CCF may rupture the ICA in conditions like an aneurysm. Moreover, neurosurgeries approaching the ACP may demand excess drilling if an incomplete CCF is present, which in turn can damage the ICA and surrounding intracranial structures. So, intricate anatomical knowledge about the uncommon variant, bilateral incomplete CCF, is of paramount importance to neurosurgeons to avoid injury to the associated structures during interventions and to radiologists to improve the interpretation of radiographs.
